# Clinical and Genetic Characteristics of Early and Advanced Gastric Cancer

**DOI:** 10.3390/cimb46020077

**Published:** 2024-02-01

**Authors:** Gi Won Ha, Hong Pil Hwang, Yong Gon Cho, Joonhong Park

**Affiliations:** 1 Department of Surgery, Jeonbuk National University Medical School and Hospital, Jeonju 54907, Republic of Korea; acts29@jbnu.ac.kr (G.W.H.); h2p@jbnu.ac.kr (H.P.H.); 2 Department of Laboratory Medicine, Jeonbuk National University Medical School and Hospital, Jeonju 54907, Republic of Korea; 3 Research Institute of Clinical Medicine of Jeonbuk National University-Biomedical Research Institute of Jeonbuk National University Hospital, Jeonju 54907, Republic of Korea; 4 Department of Laboratory Medicine, Daejeon St. Mary’s Hospital, Daejeon 34943, Republic of Korea

**Keywords:** gastric cancer, Oncomine focus assay, *PIK3CA*, *KRAS*, *ERBB2*, microsatellite instability

## Abstract

Gastric cancer (GC) persists as the fourth most prevalent cause of global cancer-related mortality, presenting a challenge due to the scarcity of available therapeutic strategies. Precision medicine is crucial not only in the treatment but also in the management of GC. We performed gene panel sequencing with Oncomine focus assay comprising 52 cancer-associated genes and MSI analysis in 100 case-matched gastric cancer cases. A comprehensive analysis of clinical and genetic characteristics was conducted on these genetic results and clinicopathological findings. Upon comparison of clinicopathological characteristics, significant differences between early gastric cancer (EGC) and advanced gastric cancer (AGC) were observed in tumor location (*p* = 0.003), Lauren classification (*p* = 0.015), T stage (*p* = 0.000), and N stage (*p* = 0.015). The six most frequently mutated genes were *PIK3CA* (29%, 10/35), *ERBB2* (17%, 6/35), *KRAS* (14%, 5/35), *ALK* (6%, 2/35), *ESR1* (6%, 2/35), and *FGFR3* (6%, 2/35). Regarding genetic variation, there was a tendency for the N stage to be higher in GC patients with mutated genes (*p* = 0.014). The frequency of mutations in GC patients was statistically significantly higher in AGC (n = 24) compared to EGC (n = 11) (odds ratio, 2.792; 95% confidence interval, 1.113 to 7.007; *p* = 0.026). Six of the ten GC patients carrying mutated genes and exhibiting MSI were classified into intestinal-type and undifferentiated GC, with the location of the tumor being in the lower-third. Among these patients, five harbored mutated *PIK3CA*, while the remaining patient had a mutation in *ALK*. Conclusions: AGC patients more frequently exhibited alterations of *PIK3CA*, *KRAS*, and *ERBB2* as somatic oncogenic drivers, and displayed a higher prevalence of cumulative genetic events, including increased rates of *PIK3CA* mutations, enhanced detection of immunotherapy biomarkers, and mutations of the *ESR1* gene.

## 1. Introduction

Gastric cancer (GC) is a complex ailment influenced by various factors, encompassing both environmental and genetic elements [1]. Global statistics indicate that GC ranks as the fourth most common cause of cancer-related deaths, with a median survival rate of less than 12 months for advanced-stage cases [2]. According to the Korea Central Cancer Registry, 29,279 individuals were newly diagnosed with GC, making it the fourth leading cause of cancer-related deaths in the Republic of Korea. This accounts for 12.0% of the total cancer incidence in the country [3]. As per a nationwide survey conducted in 2019 by the Korean Gastric Cancer Association, focusing on surgically treated GCs [4], there was a consistent rise in the proportion of upper-third tumors, escalating from 11.2% in 1995 to 20.9% in 2019. Additionally, the percentage of early GC (EGC) cases increased from 57.7% in 2009 to 63.6% in 2019, whereas advanced GC (AGC) cases are decreasing in proportion accordingly.

Advancements in genomic technologies, such as next-generation sequencing (NGS), have enabled researchers to comprehensively analyze the somatic mutation profiles of GCs at various stages [5,6,7]. This knowledge contributes to the ongoing efforts to unravel the genetic mechanisms driving GC progression and aids in the development of targeted therapies tailored to the genetic characteristics of the tumor. It is essential to note that the field of cancer genomics is continually evolving, and ongoing research may provide more insights into the nuanced differences in somatic mutation status between early and advanced GC. Specific genes may be more commonly mutated in GC, and the identification of these differences can have implications for diagnosis, prognosis, and treatment decisions. For example, certain mutations may confer sensitivity or resistance to particular therapies, making the understanding of somatic mutation status crucial for personalized cancer treatment strategies. Numerous studies have extensively explored genetic biomarkers in GC to unveil a broad spectrum of recognition patterns in this domain. Key indicators for the development of GC include *ERBB2* expression, factors governing apoptosis, regulators of the cell cycle, elements influencing cell membrane properties, multidrug resistance proteins, and microsatellite instability (MSI) [8,9]. Particularly, the amplification of *ERBB2* is more prevalent in the intestinal histologic subtype GC compared to the diffuse subtype. This amplification is not correlated with gender and age but is associated with poor survival among GC patients [10]. On the other hand, the occurrence of mutations in signaling pathways is a pivotal event, and one such affected pathway is the phosphoinositide 3-kinase (PI3K)/AKT/mammalian target of the rapamycin pathway (PI3K/AKT/mTOR pathway) [11]. A critical step in this pathway involves the generation of phosphatidylinositol-3,4,5-triphosphate (PIP3), facilitated by PI3K3. This pathway holds significance in cancer-related cellular functions, including proliferation, catabolism, cell adhesion, apoptosis, and autophagy [12]. Mutations in the p53 gene manifest in the early stages of GC, and their frequency tends to escalate as cancer advances. Patients positive for TP53 mutations are additionally classified as one of the GC subtypes [13]. On the other hand, MSI serves as a crucial indicator of DNA mismatch repair deficiency, contributing to an increased accumulation of genetic alterations in GC. In MSI-positive patients, there does not exist a high prevalence of targeted mutations; however, some have been identified in *EGFR*, *ERBB2*, *ERBB3*, and *PIK3CA* genes. GC cases exhibiting high MSI levels may experience long-term survival, irrespective of the positive resection margin status [14].

In this study, we investigated the clinical and genetic characteristics in 100 case-matched GC cases. We examined and contrasted the genetic profiles of GC tissue samples based on GC subtypes (EGC and AGC), tumor location, Lauren classification, differentiation, and tumor–node–metastasis (TNM) stage, as well as mutation landscape and MSI status.

## 2. Materials and Methods

### 2.1. Sample Preparation and DNA Extraction

One hundred formalin-fixed paraffin-embedded (FFPE) GC samples, comprising 50 EGC and 50 AGC cases, were collected from Daejeon St. Mary’s Hospital in Daejeon, Republic of Korea. Recruitment involved matching individuals based on mean age (65.5 years, range 34–84 years in EGC; 65 years, range 39–81 years in AGC) and gender (31 males in EGC; 36 males in AGC) for both groups to ensure comparable demographics. The percentage of tumor cells in relation to other cell types was assessed on a single hematoxylin and eosin (H&E)-stained tumor section by a board-certified pathologist through microscopic visual examination. An area with a minimum tumor cell content exceeding 20% was specifically chosen for microdissection, facilitating the enrichment and extraction of tumor-specific DNA. Genomic DNA was isolated from 100 FFPE GC samples using the RecoverAll Total Nucleic Acid Isolation KIT (Thermo Fisher Scientific, Waltham, MA, USA) according to the manufacturer’s instructions. Amplifiable genomic DNA was determined by fluorometric quantitation using a Qubit 2.0 Fluorometer with Qubit dsDNA HS Assay *KIT*s and the TaqMan RNase P Detection Reagents KIT (Thermo Fisher Scientific) according to the manufacturer’s protocols and was considered appropriate when the nucleic acid concentration was >30 ng/μL.

### 2.2. Library Preparation and Gene Panel Sequencing

All samples in this study underwent analysis using the commercially available Oncomine focus assay (OFA) platform. The genes targeted in this panel are meticulously chosen biomarkers sourced from expertly curated cancer genomics data [15]. Library preparations were performed using the OFA, Chef-Ready Library (Thermo Fisher Scientific), and an Ion Chef instrument (Thermo Fisher Scientific), following the manufacturer’s instructions. A total of 10 ng of DNA was utilized in the process. Then, DNA libraries were normalized to 100 pM using the Ion Chef instrument and combined before templating. Six DNA samples were loaded onto a 318™ V2 chip (Thermo Fisher Scientific) and subsequent ion semiconductor sequencing was conducted on an Ion PGM™ Dx instrument (Thermo Fisher Scientific).

### 2.3. Bioinformatic Analysis

During each sequencing run, several quality metrics were evaluated, including chip loading density, total number of reads, clonality percentage, adapter dimer percentage, low-quality percentage, read length, and alignment of reads to the hg19 human reference genome (Torrent server, version 5.12, Thermo Fisher Scientific). The “Coverage Analysis” plugin was applied to assess the quality of sample sequencing. Stringent validation criteria were set for each sample, requiring a minimum of 400,000 reads, 98% of amplicons with a minimal sequencing depth of 500×, 90% of reads located within the target region boundaries, 80% of amplicons being read from end-to-end, and 90% of amplicons being read without strand bias. The primary analysis for DNA variant annotation was conducted using Torrent Server™ v 5.12 (Thermo Fisher Scientific), followed by additional analysis on Ion Reporter™ Server, which hosts informatic tools, specifically Ion Reporter™ Software v5.10, for variant analysis, filtering, and annotations. Default parameters from the “Oncomine Focus w2.4-DNA-Single Sample” automatic workflow were adjusted: variants were reported with allele view, complex variants were permitted, down-sampling to coverage was set to 5000 reads, and variants were generated at a minimum variant allele frequency (VAF) of 0.03 with preconfigured parameter settings (Oncomine Variants 5% CI somatic CNV ploidy ≥ gain of 2 over normal) utilized. The hotspot file was also modified to include additional hotspot mutations, reporting minor variants within *BRAF*, *KRAS*, and NRAS genes. The minimum VAF detection value was lowered to 0.02 for positions of theranostic interest within *BRAF*, *EGFR*, *KRAS*, and *NRAS* genes. Public databases were used to identify known germline variants. Genetic variants identified were interpreted by board-certified laboratory geneticists and categorized as either (likely) pathogenic, variant of uncertain significance (VUS), or (likely) benign according to the American College of Medical Genetics and Genomics standards. In assessing mutation frequencies of individual genes, ‘(likely) pathogenic’ and VUS were counted as presumptive mutations; ‘benign’ and ‘likely benign’ variants were excluded.

### 2.4. Determination of Microsatellite Instability Status

A single multiplex polymerase chain reaction (PCR) reaction (Genetree Research, Seoul, Republic of Korea) amplified five microsatellite loci (NR27, NR21, NR24, BAT25, and BAT26), using genomic DNA extracted from GC and normal control samples. The PCR products were subsequently analyzed using capillary electrophoresis with 3500xL Dx Genetic Analyzer (Applied Biosystems, Foster City, CA, USA). MSI status was determined based on the data obtained from the sequencer by independent board-certified laboratory geneticists. Interpretation criteria were as follows: instability at more than one locus was classified as MSI-H, instability at a single locus was classified as MSI-L, and no instability at any locus was classified as MSS.

### 2.5. Statistical Analysis

Data were expressed as means ± standard deviations (SD) or count (percentage), as appropriate. A Student’s *t*-test was employed for continuous variables, while a Pearson’s chi-squared or Fisher’s exact test was conducted for categorical variables to assess the distinctions between EGC and AGC. The backward stepwise procedure was performed to construct a multiple logistic regression model, equating the relationships of clinical and genetic characteristics between two groups. A nonsignificant result of a Hosmer and Lemeshow test supported the goodness-of-fit of our model. All statistical calculations were carried out using MedCalc statistical software version 19.8.3 (MedCalc Software, Ltd., Ostend, Belgium). A significance level of *p* < 0.05 was considered to indicate a statistically significant difference.

## 3. Results

### 3.1. Clinicopathological Characteristics

Primary tumors originated from various locations of the stomach: 18 from the upper-third, 18 from the middle-third, 58 from the lower-third, and 6 involving the whole stomach. All histopathologic diagnoses underwent independent reviews by two board-certified pathologists, and in all cases, their assessments were concordant. According to the Lauren classification, 51 cases were categorized as intestinal-type, 27 as diffuse-type, and 22 as mixed-type. Among these patients, 50 were in stage T1, 9 in stage T2, 10 in stage T3, and 31 in stage T4. In addition, 35 were in stage N1, 16 in stage T2, 19 in stage T3, and 30 in stage T4, while 98 were in stage M0 and 2 in stage M1. On the other hand, upon comparison of clinicopathological characteristics between EGC and AGC, significant differences were observed in tumor location (*p* = 0.003), Lauren classification (*p* = 0.015), T stage (*p* = 0.000), and N stage (*p* = 0.015). The results of the logistic regression showed that the frequency of tumors occurring in the upper-third location was 0.9 times lower in EGC (*p* = 0.001). Additionally, the diffuse-type of Lauren classification was 0.8 times lower in EGC (*p* = 0.001). The comparison of clinicopathological characteristics between EGC and AGC is presented in Table 1.

### 3.2. Landscape of Somatic Mutations

**Figure 1:**
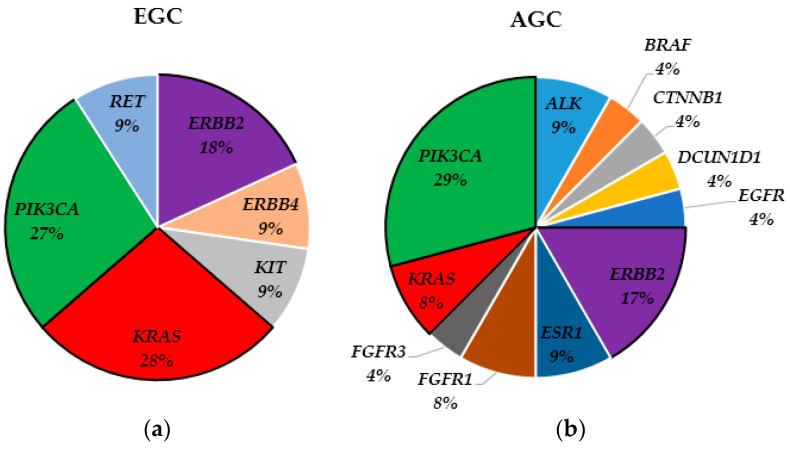
Pie charts showing the frequencies of the gene mutations. (**a**) Frequencies of the different gene mutations in early gastric cancer (EGC). (**b**) Frequencies of the different gene mutations in advanced gastric cancer (AGC). The three most frequently mutated genes, *PIK3CA* (filled with green), *ERBB2* (filled with violet), and *KRAS* (filled with red), are highlighted with a black border.

All variants were identified with over 99% confidence, determined by allele frequency and amplicon coverage. The average sequencing depth of coverage was greater than 500, and the analytic sensitivity was >5% variant allele frequency. Gene panel sequencing using OFA achieved an average coverage of 638× for tumor genomes with a Q30 score of 0.92. We used Ion Reporter software v5.10 to define significantly mutated genes in 100 GC patients, and identified 35 pathogenic mutations or VUS of 14 genes in 28 GC patients. The six most frequently mutated genes were *PIK3CA* (29%, 10/35), *ERBB2* (17%, 6/35), *KRAS* (14%, 5/35), *ALK* (6%, 2/35), *ESR1* (6%, 2/35), and *FGFR3* (6%, 2/35). We found that *PIK3CA* tended to mutate in patients whose tumor was located in the lower-third (7/10), classified as intestinal-type GC (6/10), and undifferentiated GC (6/10); however, there was no significant difference in frequency compared to other mutant patients. In addition to common mutations, various genes exhibited mutations in EGC (n = 3; *ERBB4*, *KIT*, and *RET*) and AGC (n = 8; *ALK*, *BRAF*, *BRAF*, *DCUN1D1*, *EGFR*, *ESR1*, *FGFR1,* and *FGFR3*). AGC exhibited a more diverse mutation landscape. Regarding genetic variation, there was a tendency for the N stage to be higher in GC patients with mutated genes (*p* = 0.014). A comparative analysis of significantly mutated genes was conducted between EGC and AGC. As a result, patients with AGC (n = 24) exhibited a statistically higher prevalence of mutations compared to those with EGC (n = 11) (*p* = 0.026). Interestingly, the commonly mutated genes such as *ERBB2*, *KRAS*, and *PIK3CA* that were observed in EGC were also found to be common in AGC. While *PIK3CA* was the most frequently observed in both groups, *KRAS* showed a higher prevalence in EGC (3 in EGC; 2 in AGC), and *ERBB2* exhibited a higher prevalence in AGC (4 in AGC; 2 in EGC). However, these differences did not reach statistical significance. Specifically, seven cases of GC with mutated *PIK3CA* and unstable MSI were located in the lower-third of the tumor. Except for three common mutations, more various kinds of mutated genes were observed: *ERBB4*, *KIT*, and *RET*) (Figure 1).

### 3.3. Microsatellite Instability Status

**Figure 2:**
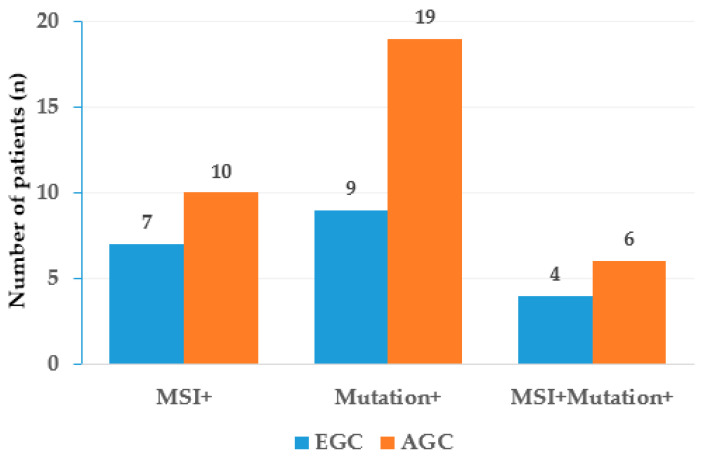
Mutation frequencies and microsatellite instability (MSI) status in early gastric cancer (EGC) and advanced gastric cancer (AGC). The figure demonstrates the proportion of samples in EGC and AGC bearing at least one mutation in a given gene. MSI+, samples with unstable MSI; Mutation+, samples carrying mutated genes identified by Oncomine focus assay; MSI+Mutation+, samples with unstable MSI harboring mutated genes identified by Oncomine focus assay.

Out of 100 GC patients, unstable MSI was detected in 17 individuals. Specifically, seven patients were positive in EGC, and ten patients were positive in AGC. However, there was no significant difference in the frequency of unstable MSI between the two groups (Figure 2).

Out of the ten GC patients carrying mutated genes and exhibiting MSI, six were classified into intestinal-type GC and undifferentiated GC, with the location of the tumor being in the lower-third. Among these patients, five harbored mutated *PIK3CA*, while the remaining patient had a mutation in *ALK*. The MSI status and gene panel sequencing results in 35 out of 100 case-matched GC cases are presented in Table 2.

## 4. Discussion

In high-risk countries, particularly East Asian nations, there is a notable emphasis on the diagnosis of EGC, leading to the implementation of population screening programs for asymptomatic patients. This proactive approach has resulted in a significant rise in the proportion of EGC cases in recent decades, particularly in Japan and South Korea [16]. EGC is differentiated from AGC based on the extent of gastric wall invasion. EGC is characterized as a carcinoma confined to the mucosa (T1a) or submucosa (T1b), irrespective of lymph node involvement. In the TNM classification, EGC is categorized as T1, while AGC encompasses T2 to T4. From an endoscopic perspective, it is feasible to anticipate gastric cancer as EGC by assessing microscopic appearance, mucus pattern, and vascular pattern. Additional diagnostic tools such as endoscopic ultrasound and abdominal tomography contribute to the clinical differentiation between EGC and AGC [17]. In this investigation, we conducted cancer gene panel sequencing to compare the clinical and genetic characteristics between EGC and AGC. The findings revealed a higher number of mutated genes and increased MSI status in AGC when compared to EGC. Our study aligns with previous research [14,18,19], identifying recurrent *PIK3CA*, *ERBB2*, *KRAS*, *ALK*, *ESR1*, and *FGFR3* mutations in both EGC and AGC. This implies that these mutations could potentially serve as key drivers in the early stages of gastric tumorigenesis. The presence of genetic heterogeneity plays a pivotal role in cancer evolution, leading to phenotypic diversity. Genomic instability stands out as a major contributor to genetic heterogeneity in cancer. Tumors, influenced by genetic and epigenetic changes, as well as modified tumor microenvironments, consist of varied subclones exhibiting diverse genetic and phenotypic traits. The collaboration among these diverse subclones, facilitated through paracrine signaling, cell–cell contact, and microenvironment alterations, provides them with a fitness advantage throughout the progression of the tumor [20]. Unlike our study, in a Spanish GC cohort, EGC exhibited distinctions from advanced GC in various aspects such as location (predominantly antrum and incisura in 76% vs. 38%, *p* = 0.01) [21]. EGC was predominantly located in the lower-third of the stomach (antrum and angular incisure) compared to AGC.

The comparison of clinical and genetic characteristics in GC patients is crucial for understanding disease progression and tailoring effective treatment strategies. Comprehensive genomic analyses, such as NGS, can reveal the specific mutations and alterations in both early and advanced stages, guiding targeted therapy choices. The mutation landscape varies between EGC and AGC. Specific genes may be more frequently mutated in advanced stages, influencing treatment decisions. Molecular markers associated with prognosis, such as certain gene mutations or expression profiles, may differ between EGC and AGC. *ERBB2* amplification or overexpression may be more prevalent in AGC, affecting the eligibility for HER2-targeted therapies. On the other hand, *PIK3CA* mutations were predominantly observed in the upper-third of the stomach and exhibited solely intestinal histology. They exhibit a strong association with the MSI genetic subgroup, indicating a poorer prognosis compared to GC patients with wild-type *PIK3CA* [19]. Furthermore, a significant majority, specifically 52.6%, of *PIK3CA*-mutant GCs belonged to the chromosomal instability (CIN) or genomic stable subtype. This subgroup displayed decreased PD-L1 expression and lower stromal tumor-infiltrating lymphocytes (TIL) when contrasted with the Epstein–Barr virus-positive (EBV+) and MSI-H subtypes. Strikingly, all patients within the CIN or GS subtype exhibited a lack of response to immune checkpoint inhibitor treatment [22].

On the other hand, in cancer research, especially in the context of gastric cancer, researchers often consider genetic heterogeneity when selecting tumor samples for gene analysis. The genetic makeup of tumors can vary between different regions within the same tumor, leading to intra-tumoral heterogeneity. To account for this, researchers may choose to analyze multiple tumor foci, including samples from both the core and the invasive front of the gastric tumor. This approach aims to capture the diversity of genetic alterations that may exist within different areas of the tumor. It is indeed possible that a tumor sample taken from the core of a gastric tumor shows a *PIK3CA* mutation, while a sample from the invasive front exhibits a wild-type status for the gene. This genetic heterogeneity is an important consideration in understanding the complexity of gastric cancer and its potential implications for treatment strategies [20]. Researchers may employ techniques such as multi-region sampling or single-cell sequencing to better characterize the genetic heterogeneity within tumors. By doing so, they aim to provide a more comprehensive and accurate representation of the genetic landscape of gastric cancer [23,24]

MSI constitutes approximately 15–30% of GCs, with the majority falling under the category of intestinal-type, which has been linked to certain demographic and clinical characteristics. Patients with MSI GCs are often older (≥65 years), more frequently female, and exhibit tumors located in the middle/lower gastric body [25]. Furthermore, they tend to have less frequent lymph node involvement and a lower inclination to invade serosal layers. MSI GC patients are commonly diagnosed at earlier disease stages (TNM stage I or II) and classified as Borrmann type I or II [26]. Histologically, these tumors typically feature highly pleomorphic cells organized in distinctive growth patterns, association with mucinous GC or mucin 6 positivity, and prominent infiltration of lymphoid cells [27]. However, the MSI subgroup appears to be diverse, suggesting that other genetic factors might be influential for these specific patients [28,29]. In EGC, which involves tumors confined to the mucosa or submucosa without spreading beyond the stomach wall, the somatic mutation landscape may differ from that of AGC, where the cancer has progressed to deeper layers of the stomach wall or metastasized to other organs. In EGC, which refers to tumors that are confined to the mucosa or submucosa and have not spread beyond the stomach wall, there may be variations in MSI status compared to AGC, where the cancer has progressed to deeper layers of the stomach wall or beyond [30]. Studies suggest that MSI-high (MSI-H) tumors, characterized by a high level of microsatellite instability, may be more prevalent in certain subtypes of GC. These tumors exhibit a higher mutation rate due to impaired DNA repair mechanisms, and this can have implications for prognosis and treatment response. On the other hand, MSI-low (MSI-L) or microsatellite stable (MSS) tumors are associated with a lower frequency of replication errors. Understanding the MSI status in both early and advanced stages of GC is crucial for tailoring treatment strategies, predicting patient outcomes, and advancing our knowledge of the genetic mechanisms underlying gastric carcinogenesis. Further research is needed to explore the nuances of MSI status in different stages of GC and its clinical implications [31].

This study presents several limitations. Firstly, clinical outcome data were not accessible to establish correlations between identified mutations and treatment response or survival, as this falls beyond the scope of the current study. Furthermore, a majority of the patients did not have an estimated stage for the latest illness, even if the tumor submitted originated from an FFPE sample generated from surgical tissue collected during the initial diagnosis. However, whether patients presented with de novo metastatic disease and their history of previous lines of therapy remain unclear. It is essential to note that selection bias could have impacted our reported genetic results, and the differences observed by age- and gender-matched case groups might not accurately represent the broader population of GC patients. No copy number variations (CNV) were detected in this study. It is important to note that CNV results obtained through NGS analysis may be prone to false negatives, and these findings were not confirmed using immunohistochemistry. Even though the performance of the OFA panel, which targets various mutations in 52 genes represented in the OFA panel, was assessed using a highly multiplexed test panel [32], the TP53 gene was not represented in the OFA panel. In recent study [33], the mutational analysis of TP53_RTAS_ may enhance the identification of patients who are likely to derive the greatest benefit from Ramucirumab therapy. Additionally, the evaluation of TP53RTAS analysis could be extended to patients with metastatic adenocarcinomas in other solid tumors characterized by frequent TP53 mutations and where anti-VEGF therapy is commonly used. We are currently unable to assess the correlation between mutations and germline mutations when there is no sequenced matched normal tissue. This lack of information may contribute to the observed differences in mutation frequencies between EGC and AGC.

## 5. Conclusions

In summary, this comparative evaluation of clinical and genetic characteristics from 100 case-matched GCs revealed distinct genetic trends based on GC subtype and MSI status. Notably, AGC patients more frequently exhibited *PIK3CA*, *KRAS*, and *ERBB2* as somatic oncogenic drivers, and displayed a higher prevalence of cumulative genetic events, including increased rates of *PIK3CA* mutations, enhanced detection of immunotherapy biomarkers, and mutations of the *ESR1* gene. Our findings contribute valuable information to the growing body of literature exploring tumor differences between EGC and AGC, potentially informing optimal treatment strategies for each GC subtype. More comprehensive studies are required to investigate the correlation between the genetic pathways associated with *PIK3CA* and the status of MSI. Further comprehensive efforts to investigate the clinical and genetic distinctions in GC patients are required for advancing precision medicine in GC, aiding in early detection, and optimizing treatment approaches based on the unique characteristics of each stage.

## Figures and Tables

**Table 1:** Comparison of clinicopathological characteristics in 100 case-matched gastric cancer cases.

Variable	EGC (n = 50)	AGC (n = 50)	*p* Value
Age (year), mean (range)	65.5 (34–84)	65 (39–81)	
Gender (n), Male/Female	31/19	36/14	0.198
Tumor location (n)			0.003
Upper-third	6	12	
Middle-third	16	2	
Lower-third	26	32	
Whole	2	4	
Lauren classification (n)			0.015
Intestinal	21	30	
Diffuse	12	15	
Mixed	17	5	
Differentiation (n)			0.689
Differentiated	25	27	
Undifferentiated	25	23	
T stage (n), T1/T2/T3/T4	50/0/0/0	0/9/10/31	0.000
N stage (n), N0/N1/N2/N3	25/5/7/13	10/11/12/17	0.015
M stage (n), M0/M1	50/0	48/2	0.153
H. pylori infection (n)	2	5	0.240
Mutation status (n)			
MSI-positive	7	10	0.424
Mutation-positive	9	19	0.026
MSI- and mutation-positive	4	6	0.505

EGC, early gastric cancer; AGC, advanced gastric cancer; MSI, microsatellite instability.

**Table 2:** MSI status and gene panel sequencing results in 35 out of 100 case-matched gastric cancer cases.

Sample	MSI	Gene	Reference ID	Base Change	AA Change	Class
EG01	Neg	*KRAS*	NM_004985.5	c.57G>C	p.Leu19Phe	Pathogenic
EG04	Pos	Negative				
EG08	Pos	Negative				
EG10	Pos	*ERBB2*	NM_004448.4	c.2524G>A	p.Val842Ile	VUS
		*ERBB4*	NM_005235.3	c.2804A>G	p.Lys935Arg	VUS
EG14	Neg	*KRAS*	NM_004985.5	c.38G>A	p.Gly13Asp	Pathogenic
EG20	Pos	*ERBB2*	NM_004448.4	c.2524G>A	p.Val842Ile	VUS
		*PIK3CA*	NM_006218.4	c.3140A>G	p.His1047Arg	Pathogenic
EG21	Neg	*KIT*	NM_000222.3	c.1689A>G	p.Ile563Met	VUS
EG31	Neg	*KRAS*	NM_004985.5	c.437C>T	p.Ala146Val	Pathogenic
EG37	Neg	*PIK3CA*	NM_006218.4	c.1637A>G	p.Gln546Arg	Pathogenic
EG41	Pos	*RET*	NM_020975.4	c.2636delA	p.Asn879Thrfs*4	Likely Pathogenic
EG48	Pos	*PIK3CA*	NM_006218.4	c.1637A>G	p.Gln546Arg	Pathogenic
EG49	Pos	Negative				
AG02	Pos	Negative				
AG03	Neg	*ERBB2*	NM_004448.4	c.2524G>A	p.Val842Ile	VUS
AG09	Pos	Negative				
AG10	Pos	Negative				
AG11	Pos	*EGFR*	NM_005228.5	c.2227G>A	p.Ala743Thr	VUS
		*ESR1*	NM_000125.4	c.1652C>T	p.Ala551Val	VUS
		*KRAS*	NM_004985.5	c.38G>A	p.Gly13Asp	Pathogenic
AG13	Pos	*ALK*	NM_004304.5	c.1999G>A	p.Gly667Arg	VUS
		*FGFR3*	NM_000142.5	c.274delC	p.Gln92Serfs*6	VUS
AG14	Neg	*ALK*	NM_004304.5	c.4061G>T	p.Cys1354Phe	VUS
AG15	Pos	*PIK3CA*	NM_006218.4	c.325GAA	p.Glu110del	Pathogenic
AG16	Pos	*PIK3CA*	NM_006218.4	c.323G>A	p.Arg108His	Pathogenic
AG17	Neg	*ERBB2*	NM_004448.4	c.3149C>T	p.Ser1050Leu	VUS
AG18	Neg	*DCUN1D1*	NM_020640.4	c.19T>G	p.Ser7Ala	VUS
AG23	Pos	*PIK3CA*	NM_006218.4	c.325GAA	p.Glu110del	Pathogenic
AG27	Neg	*ERBB2*	NM_004448.4	c.2033G>A	p.Arg678Gln	Pathogenic
AG28	Neg	*PIK3CA*	NM_006218.4	c.1633G>A	p.Glu545Lys	Pathogenic
AG29	Neg	*ERBB2*	NM_004448.4	c.3110C>T	p.Pro1037Leu	VUS
AG32	Pos	Negative				
AG34	Neg	*BRAF*	NM_001904.4	c.98C>T	p.Ser33Phe	Pathogenic
AG38	Neg	*FGFR1*	NM_023110.3	c.2266C>T	p.Arg756Cys	VUS
AG40	Neg	*KRAS*	NM_004985.5	c.34G>A	p.Gly12Ser	Pathogenic
		*PIK3CA*	NM_006218.4	c.1633G>A	p.Glu545Lys	Pathogenic
AG44	Neg	*FGFR3*	NM_000142.5	c.274delC	p.Gln92Serfs*6	VUS
		*PIK3CA*	NM_006218.4	c.1390T>G	p.Ser464Ala	VUS
AG46	Pos	*PIK3CA*	NM_006218.4	c.1633G>A	p.Glu545Lys	Pathogenic
AG49	Neg	*BRAF*	NM_004333.6	c.1780G>A	p.Asp594Asn	Pathogenic
AG50	Neg	*ESR1*	NM_000125.4	c.1664G>A	p.Arg555His	VUS

MSI, microsatellite instability; Pos, positive; Neg, negative; EG, early gastric cancer; AG, advanced gastric cancer; VUS, variant of uncertain significance.

## Data Availability

Data are contained within the article.
